# Cross-Species Analysis of Glycosaminoglycan Binding Proteins Reveals Some Animal Models Are “More Equal” than Others

**DOI:** 10.3390/molecules24050924

**Published:** 2019-03-06

**Authors:** Eric D. Boittier, Neha S. Gandhi, Vito Ferro, Deirdre R. Coombe

**Affiliations:** 1 School of Chemistry and Molecular Biosciences, the University of Queensland, Brisbane, QLD 4072, Australia; eric.boittier@uq.net.au (E.D.B.); v.ferro@uq.edu.au (V.F.); 2 School of Mathematical Sciences and Institute for Health and Biomedical Innovation, Faculty of Science and Engineering, Queensland University of Technology, Brisbane, QLD 4000, Australia; 3 School of Pharmacy and Biomedical Sciences, Curtin Health Innovation Research Institute, Faculty of Health Sciences, Curtin University, Perth, WA 6102, Australia

**Keywords:** antithrombin, glycosaminoglycans, chemokines, heparin binding proteins, eotaxin, IL-8, molecular modelling

## Abstract

Glycosaminoglycan (GAG) mimetics are synthetic or semi-synthetic analogues of heparin or heparan sulfate, which are designed to interact with GAG binding sites on proteins. The preclinical stages of drug development rely on efficacy and toxicity assessment in animals and aim to apply these findings to clinical studies. However, such data may not always reflect the human situation possibly because the GAG binding site on the protein ligand in animals and humans could differ. Possible inter-species differences in the GAG-binding sites on antithrombin III, heparanase, and chemokines of the CCL and CXCL families were examined by sequence alignments, molecular modelling and assessment of surface electrostatic potentials to determine if one species of laboratory animal is likely to result in more clinically relevant data than another. For each protein, current understanding of GAG binding is reviewed from a protein structure and function perspective. This combinatorial analysis shows chemokine dimers and oligomers can present different GAG binding surfaces for the same target protein, whereas a cleft-like GAG binding site will differently influence the types of GAG structures that bind and the species preferable for preclinical work. Such analyses will allow an informed choice of animal(s) for preclinical studies of GAG mimetic drugs.

## 1. Introduction

Glycosaminoglycans (GAGs) are natural heteropolysaccharides that are composed of repeating disaccharide units consisting of a uronic acid linked to an amino sugar, either of which can be sulfated or non-sulfated [1]. They are of varying size and sulfation patterns and are present in every mammalian tissue. GAGs play important roles in both normal physiological processes and pathological conditions [2]. Recently, experimental biophysical studies have revealed that GAGs such as heparin and heparan sulfate can affect cell properties and functions by acting directly on cell receptors or via interactions with growth factors and chemokines. As the role(s) of GAGs in many essential biological processes has become appreciated, associated structural studies of GAGs complexed with proteins involved in the various biological processes has led to the exploration of the therapeutic potential of GAG mimetics. GAG mimetics are anionic compounds that mimic the structure of GAGs and bind to the same, or overlapping, site(s) as natural heparin on their protein ligands. By their design, some of these mimetics have overcome issues such as binding specificity and potency; for example, some mimetics are conjugated to complementary chemical moieties, and/or the structure of their backbone may dictate a sulfation pattern that limits their binding to a subset of heparin binding proteins [3,4]. The translation of a heparin mimetic from basic research into clinical studies requires preclinical testing in animal disease models and toxicity testing in several animal species. Frequently, rodents are used for the disease model without any understanding as to whether the heparin binding site on the protein primarily targeted by the mimetic, is similar in both humans and rodents. This is an important issue because the choice of the animal species used could have implications as to the assumed efficacy of the mimetic. For example, if the mimetic has been carefully designed to slot into a binding site on a human protein, and this binding site is not mirrored in the amino acid composition and/or electrostatic potential of that protein in the animal model used, it is likely the efficacy of the mimetic will be compromised, and the data obtained unreliable.

Heparin binding proteins are found in several therapeutic areas, with coagulation and thrombosis, cancer, and inflammatory diseases being those most commonly cited. Here we have examined the binding sites on six heparin binding proteins from different therapeutic areas in an effort to understand the extent to which GAG binding sites are conserved across mammals. The heparin binding proteins chosen were the enzymes antithrombin III (AT) and heparanase, and the chemokines RANTES (Regulated on Activation Normal T cell Expressed and Secreted; CCL5), interleukin-8 (IL-8; CXCL8) eotaxin-1 (CCL11) and platelet factor 4 (PF4; CXCL4). These proteins were chosen because they have been the targets for drug discovery or, in the case of PF4-heparin complexes, they trigger potential toxicity issues in humans.

Fondaparinux, a rationally designed heparin mimetic has been approved for prophylaxis and treatment of venous thromboembolism [5,6]. Fondaparinux, the fully synthetic methyl glycoside of the AT activating pentasaccharide sequence in heparin, binds AT and accelerates its inhibition of factor Xa to act as a potent anticoagulant. The three-dimensional X-ray crystal structure of fondaparinux with AT has been solved and fondaparinux was shown to occupy the same binding site as the heparin pentasaccharide [7]. Previous studies have shown that fondaparinux and other closely related oligosaccharides have similar binding affinities (*K*_d_ in nm) for rat and human AT, but not for AT from rabbits or baboons [8].

Heparanase is an important promotor of tumor progression [9]. Heparan sulfate mimetics, modified heparins, and related polysulfated compounds such as PI-88 (muparfostat) and PG545 (pixatimod) were developed to target heparanase and so inhibit its enzymatic activity. Both PI-88 and PG545 have been tested in clinical trials involving cancer patients but PI-88 has not yet been approved while PG545 is still in clinical development [3]. These mimetics are generally well tolerated when administered to patients and have low or no anticoagulant activity via AT, but PI-88 acts as a weak anticoagulant by binding heparin cofactor II (HC-II) and so enhances HC-II inhibition of thrombin [10]. PI-88 has also been reported to trigger thrombocytopenia in some patients and this was immunologically mediated as anti-heparin-PF4 complex antibodies were detected. The development of drug-induced thrombocytopenia, a severe autoimmune response triggered by formation of antibodies recognizing heparin/heparin mimetic-PF4 complexes [11], was quite unexpected as preclinical studies in rats and monkeys provided no evidence of thrombocytopenia and no indication that the development of anti-heparin-PF4 complex antibodies could result from administering PI-88 to patients. Given these findings, we wondered as to the similarity of the PF4 heparin binding site in commonly used laboratory animals with that of human PF4.

A number of heparin mimetics are under development as novel anti-inflammatory drugs targeted at chemokines such as CCL5, CCL20, and CXCL8/IL-8. The role of chemokine-GAG interactions, and chemokine-GAG mimetic interactions in inflammation have been extensively reviewed [9,12,13]. The anti-inflammatory effects of heparin mimetics could be attributed to (i) disruption of the chemokine-GAG interaction, (ii) prevention of the interaction of chemokine with its receptor, or (iii) by stabilizing/destabilizing chemokine oligomer formation by binding to multiple sites. IL-8 is the key chemokine of the CXC chemokine subfamily that directs neutrophils to inflammatory sites. GAGs are known to bind IL-8 monomers and dimers at two different sites, termed parallel or perpendicular binding. The latter spans both monomers within an IL-8 dimer whereas the former was found to be confined to a single monomer. Both binding modes have implications on how IL-8 binds its receptors CXCR1 and CXCR2 in the presence of GAGs. As amino acids involved in GAG binding are also required for receptor binding [14,15] GAG-bound IL-8 will be impaired for receptor binding.

GAGs also bind chemokines of the CC chemokine sub-family which includes the eotaxins and CCL5 [16,17]. Eotaxins and particularly eotaxin-1 (CCL11), which is the most studied, are well known for stimulating the migration of eosinophils from the small blood vessels and into tissues. Originally, eotaxin-1 was described as playing a key part in allergic airway diseases like asthma and allergic rhinitis, however, it is now appreciated that this chemokine also contributes to other diseases like inflammatory bowel disease and allergic and inflammatory skin conditions [18]. Eotaxins act via the CC chemokine receptor CCR3, which is located on the cell surface of eosinophils, basophils, mast cells, and the T helper 2 (Th2) lymphocyte subset, as well as other cell types like keratinocytes and endothelial cells [18]. CCL5 similarly mediates eosinophil recruitment, but it also mediates the migration of other leukocytes including T-cells, monocytes, basophils and NK-cells and dendritic cells by signaling through its receptors CCR1, CCR3, or CCR5 [17]. It is the interaction of CCL5 with CCR1 that is best inhibited by GAG/heparin binding to CCL5 and it is this interaction that has been targeted by heparin mimetics [13]. To date most studies on inhibiting eotaxin-CCR3 mediated eosinophil recruitment have focused on CCR3 rather than the eotaxins. This may have been because a murine study demonstrated that heparin binding to eotaxin-1 potentiated eosinophil recruitment in vivo [16]. Nevertheless, it is possible that GAG mimetics of the appropriate structure and size could act on one or more of the eotaxins to disrupt eosinophil recruitment in asthma and other eosinophilic inflammatory diseases. Many animal species have been used to study asthma etiology (e.g. mice, rats, rabbits, guinea pigs, cats, dogs, swine, cows, sheep, horses, and primates) [19]. Although commonly murine allergic airway inflammation models are used, but how the GAG/heparin binding sites on the molecular mediators of this inflammation vary from mice to man has not been well studied.

There is a great deal of literature on the activity of small molecules tested in vitro that did not translate into in vivo effects in animal models, or in humans. For example, enzymes of the aldo-keto reductase (AKR) 1C subfamily have been implicated in the progression of prostate, breast, endometrial, and leukemic cancer [20]. However, their lack of conservation in mice has meant murine models cannot be used to test the role of AKR1C in cancer. Similarly and more importantly for this paper, using molecular modelling and biophysical experiments of the immunoglobulin (Ig) superfamily protein PECAM-1, we previously reported that the basic amino acid residues at the −2 and −1-positions in the heparin binding motif (in Ig domains 2 and 3) required for heparin binding in human PECAM-1, are not well conserved in rat or murine PECAM-1 [21]. As a consequence, PECAM-1 from these rodents will not bind heparin with the same affinity as human PECAM-1. From these examples, we suggest that understanding how the heparin binding motifs in proteins being targeted for the development of GAG mimetic drugs differ, between humans and the animal species commonly used for pre-clinical studies, will be essential to avoid misinterpretation of the data generated.

Experiments such as mutagenesis, X-ray crystallography, and NMR spectroscopy coupled with molecular modelling studies have revealed the amino acids comprising the heparin binding sites on our selected proteins (Table 1). Here we use this information for a comparative analysis of protein electrostatic potentials, along with sequence and structural analyses, across a set of animal species commonly used for preclinical studies to understand how well the heparin binding sites are conserved. Such analyses have previously been applied to compare glycolytic enzymes from *Homo sapiens* with those from 10 other eukaryotic species to understand enzymatic functions, glycolytic pathways, and the role of glycolytic enzymes in transcriptional regulation and apoptosis [22]. This study revealed that data on the electrostatic potentials around the active site, and amino acid sequence similarity data of the active site, provide complementary information. Given the importance of the three-dimensional (3D) electrostatic potential for GAG binding to proteins we chose to similarly include both types of analyses in our study. We applied this approach to heparanase and AT, which have 3D structures of heparin co-crystallized with the protein. The same approach was applied to two CXC and two CCL motif chemokines. Despite the fact that all chemokines possess a highly conserved tertiary, structural fold, the heparin binding sites are distinct between the CXC and CCL families [23]. To the best of our knowledge, no computational study has compared the binding site interaction profiles of heparin binding proteins across different species.

## 2. Sequence and Structure Conservation in Heparin Binding Proteins

### 2.1. Antithrombin III (AT)

AT is a serpin that acts as a suicidal substrate inhibitor of thrombin and is central to the regulation of the blood coagulation cascade [28]. Although AT in its unbound state can inhibit thrombin, when AT is bound to heparin the rate of thrombin inhibition is enhanced by up to 3000-fold. The heparin binding domain of AT recognises a highly specific pentasaccharide sequence. Binding of the heparin pentasaccharide to AT occurs in two steps; initially a weak interaction occurs and this is followed by a local conformational change in AT that extends to the reactive centre loop (RCL) of the protein [29]. Although human AT is a basic protein, fondaparinux only binds to a restricted, specific site on the protein. The crystal structure of fondaparinux complexed with AT is shown in Figure S1A and the hydrophobic surface representation shown in Figure S1B suggests that fondaparinux binds on the surface of AT. The residues that comprise the binding site, shown in Table 1 and Figure S2, are completely conserved across almost all animal species for which there are sequences. Importantly, AT amino acids in the binding site interact with heparin by both charge/ionic interactions and hydrophobic interactions as is clear from Figure S1C. The conservation of all these residues indicates the importance of both types of interactions for heparin binding as well as the importance of heparin-AT interactions for maintaining haemostasis in response to vascular injury in mammals.

**Figure 1:**
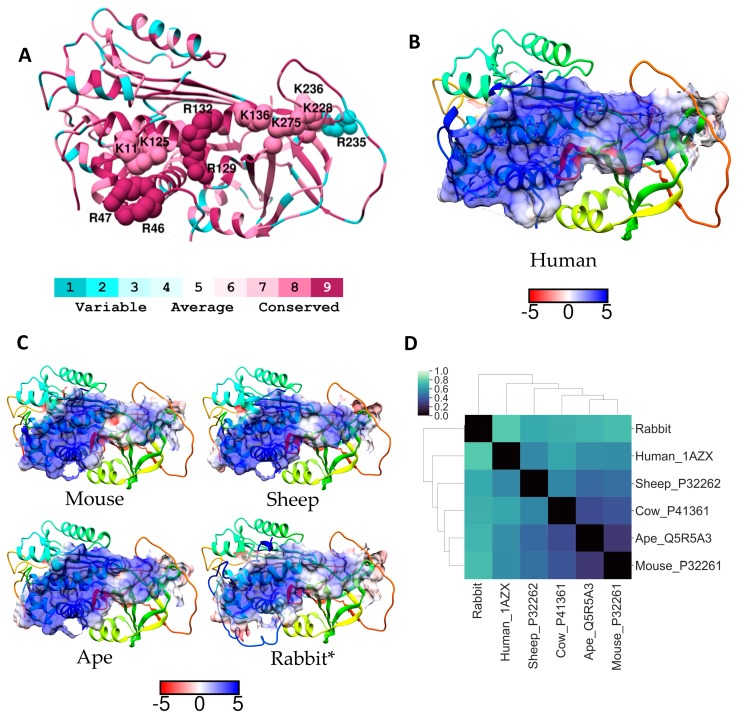
(**A**) Ribbon diagram of AT coloured according to Consurf conservation scores. Residues previously identified as important for GAG binding have been labelled and depicted as spheres (not all residues are shown for clarity). Electrostatic surface potential (blue for positive and red for negative) for (**B**) human AT and (**C**) for selected homologues of residues within 5 Å radius of GAG binding residues. Units for electrostatic potential are kT/e. (**D**) Electrostatic difference (ESD) plot showing all-pairwise comparison of the electrostatic potentials for surfaces shown in (**B**) and (**C**).

The amino acids in the region around the binding site are important for directing or orientating heparin to the correct region on the protein for binding, accordingly we have compared the electrostatic potentials of the heparin binding site and the region 5 Å around this site (GAG-binding region) on AT from four mammals with that of human AT (Figure 1B,C). Figure 1A indicates the basic residues in this GAG-binding region for the human protein. The GAG-binding region on AT from each of these species is similarly positioned on the protein surface, although the electrostatic potential in this region on human AT is more positive than that of the other proteins (Figure 1B,C). Electrostatic difference (ESD) calculations of the GAG binding region on AT, using homology models, suggested that the mouse GAG-binding region was more similar to the human binding region than the rabbit region (Figure 1D). Whereas the mouse GAG-binding region was more similar to that of the ape, cow, and sheep than human (Figure 1D).

**Figure 2:**
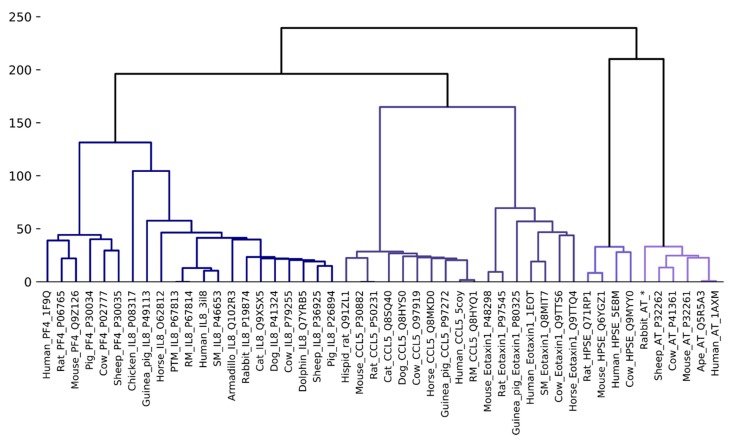
Hierarchical clustering of GAG-binding protein sequences based on percent identity.

The only homology sequence analysed that did not show conservation of all key heparin binding residues was the rabbit AT sequence, however this sequence was deduced from rabbit cDNA [30]. Our electrostatic potential analyses, similarly showed that the GAG-binding region of rabbit AT was more different from the GAG-binding region of human AT than the other species. Nevertheless, Sheffield et al. found that the rabbit AT did form complexes with human thrombin [30]. Other studies comparing the affinity of heparin binding to AT from different species, using synthetically prepared heparin oligosaccharides, found that the dissociation constant (*K*_D_) for rabbit AT was considerably higher (132 ± 10 nM) than for rat or human AT (50 ± 2.5 nM and 41 ± 8 nM respectively) [8]. Although the rat AT sequence is unavailable, data for murine AT suggest that rodent AT may be analogous to the human protein. This was supported by hierarchical clustering using the percent identity matrix of a selected group of GAG binding protein sequences (Figure 2). Full-length sequence alignments between secreted human and rabbit AT indicated conservation of residues that form a hydrophobic pocket in all serpin family members (amino acids 181–250), however, differences exist in the C-terminal region and the RCL as well as in the heparin binding region. Although these differences may alter the reliability of the rabbit as a model species for the development of heparin and low molecular weight heparin as anticoagulants, rabbits are frequently used for pharmacokinetic analyses of heparins [31].

### 2.2. Heparanase

Heparanase is an endoglycosidase that plays an integral part in the remodelling and degradation of the extracellular matrix by cleaving heparan sulfate (HS) chains on matrix and cell surface proteoglycans, which also releases a variety of growth factors [9,32]. Heparanase is primarily known for its role in tumour progression, but recent information has revealed other roles including in fibrosis, autophagy and inflammation. Heparanase cleaves HS chains into fragments of around 10–20 saccharide units by specifically recognising and cleaving internal β (1–4) glycosidic linkages between glucuronic acid (GlcUA) and N-sulfated glucosamine (GlcNS). This enzyme also cleaves heparin at the same specific linkage, giving rise to fragments ranging in size from 5 kDa to 20 kDa [33]. Importantly, specific *N*- and *O*-sulfation patterns of saccharides surrounding the GlcUA serve to restrict cleavage to a subset of GlcUAs, meaning only HS sequences of particular sulfation patterns are cleaved [24]. The larger heparin fragments resulting from heparanase cleavage indicates that the necessary sulfation patterns and the linkage that is cleaved is less frequent in heparin than in HS. The active enzyme consists of a heterodimer comprising two non-covalently linked subunits (chain A and chain B) that result from lysosomal processing by cathepsin l of pro-heparanase [33]. The catalytic activity of heparanase requires acidic pH and is maximal between pH 5.0 and 6.0, which is the pH of late endosomes or lysosomes as well as the pH of the tumour microenvironment. Thus, as well as its extracellular role heparanase acts intracellularly in the degradation and turnover of cell surface HS proteoglycans [33].

**Figure 3:**
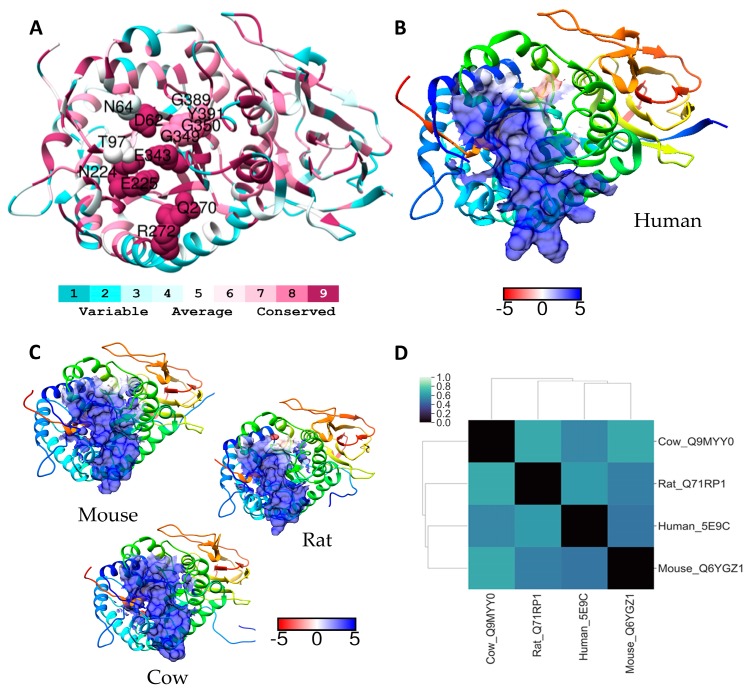
(**A**) Ribbon diagram of human heparanase coloured according to Consurf conservation scores. GAG binding around the activate site have been labelled and depicted as spheres. Electrostatic surface potential of residues within a 5 Å radius of the activate site (blue for positive and red for negative) shown for (**B**) human heparanase and (**C**) for selected homologues of residues. Units for electrostatic potential are kT/e. (**D**) Electrostatic difference (ESD) plot showing all-pairwise comparison of the electrostatic potentials for surfaces shown in (**B**) and (**C**).

The crystal structures of heparanase at pH 5.5 with semisynthetic HS oligomers, or a heparin tetrasaccharide, revealed these ligands bind heparanse within the active site cleft [24]. HS or heparin binds within a cleft of approx 10 Å in length that is lined by basic residues and contains residues Glu343 and Glu225, the nucleophile and the acid/base catalyst, respectively that are essential for the catalytic mechanism of the enzyme. HS is retained in the cleft primarily by a network of hydrogen bonds that form between the conserved non-basic amino acids in the binding site (and the GAG chain; electrostatic interactions being confined to the side chains of Lys159, Arg272 and Arg303 and the HS tetrasaccharide; see Figure S3) [24]. This dominance of hydrogen bonding is in contrast to many other GAG-protein interactions which primarily utilise ionic interactions supplemented by hydrophobic interactions. Our sequence analysis shows that catalytic residues of heparanase are completely conserved across the four species examined, and the other amino acids involved in the GAG-binding site are also conserved with the exceptions of Asn64, Thr97, Lys159, and Tyr348 in the human sequence (Figure S4). The ribbon representation of human heparanase shown in Figure 3A clearly shows how the conserved amino acids of the GAG-binding site are clustered in a central cleft structure. Of those residues that are not conserved Asn64, Thr97, and Lys159 are of particular importance for binding the GAG chain. In the human protein Asn64 forms a hydrogen bond with GlcNS and if the GlcNS is 6*O*-sulfated (both GlcNS and GlcNS(6S) are tolerated), this sulfate is well placed to interact via electrostatic interactions with Lys159, whereas hydrogen bonds from the NH of the Thr97 backbone to GlcUA were observed [24]. In the mouse and rat A chain Asn64 in the human protein is replaced by a serine, and Thr97 is replaced by an asparagine. The Lys159 in the human B chain is replaced by glutamic acid in the two rodents, suggesting that GlcNS would be the preferred structure in the chain cleaved by rodent heparanase, in contrast to GlcNS(6S) which is likely to be preferred by human heparanase. Interestingly, the sequences surrounding the residues involved in GAG binding are less well conserved, for example residues 161, 304 and 385 are not conserved, and residues 268, 273, and 274 that surround the cluster of conserved amino acids ^269^GQPR^272^ are also not conserved.

The electrostatic surface potentials for the region within 5 Å of the GAG-binding site for heparanase at pH 5.5 are shown in Figure 3B,C. The acidic pH was chosen so as to visualise the surface potentials of heparanase around the GAG-binding site in a situation where the enzyme is active, as a result more of the amino acids in this region will be protonated than would be the case at neutral pH. From this analysis it is clear that active heparanase is very positively charged in the vicinity of the binding site for all the species examined. Although there are subtle differences between species the murine protein displays the most extensive region of positive charge. The ESD plot reveals that differences between the three animal proteins and that of the human heparanase are quite high, which may suggest that recognition of the substrate by heparanase could be slightly different between species. The analysis also indicates that of the two rodent proteins examined murine heparanase is the most closely related to the human protein in this region. Although the overall sequence similarity between mice and humans is ~80% and the catalytic residues are conserved, it is likely that differences in the residues surrounding the binding region, and those differences highlighted above within the binding site, are sufficient to favour the binding and cleavage of different HS structures in murine models compared to what would occur in humans. However, GAG-mimetics currently under trial as heparanase inhibitors (PI-88 and PG545), because of their structures, are unlikely to penetrate into the GAG-binding cleft, but instead probably bind across/over the cleft surface and so block HS or heparin chains entering the cleft. Given this, our analysis suggests murine models are likely to be informative. Preclinical data on the anti-tumour effect of PI-88, were obtained in the Rip1Tag2 transgenic mouse model of pancreatic islet β-cell carcinoma, and in this system both the in vivo and ex vivo data were consistent with the anti-tumour effect of PI-88 occurring at least in part via inhibition of murine heparanase [34,35].

### 2.3. RANTES (CCL5)

CC chemokine ligand 5 (CCL5)—also known as RANTES— binds to several receptors, including CCR1, CCR3, and CCR5. CCL5 induces the migration of T cells, monocytes, basophils, eosinophils, natural killer cells and dendritic cells as part of an inflammatory response [17]. CCL5 is a highly basic, 68 amino acid protein, that is believed to recognize GAGs through the BBXB motif ^44^RKNR^47^, located on the 40s loop which is exposed on the surface of the protein [17,36]. This region was identified using ^1^H-^15^N HSQC NMR spectroscopy with the heparin disaccharide IA (ΔUA2S-α(1→4)-GlcNAc6S) as a probe. When the binding of a longer heparin fragment is examined Arg17 is included in the heparin binding site. This is particularly the case for a heparin dodecamer comprising disaccharide repeats of IdoA2S-α(1→4)-GlcNS6S), but is also the case for tetrasaccharide fragments of particular heparin structures [17]. Similar conclusions were also reached when using hexameric chondroitin sulfate (CS) as the GAG ligand. In addition to confirming the contribution of Arg17 this NMR study also convincingly demonstrated the involvement of *N*-terminal Ile15 and Leu19 in GAG binding [37]. Importantly, as seen with the heparin tetrasaccharides, different CS hexamer structures displayed different reaction patterns with CCL5 indicating that the way GAGs bind CCL5 is highly dependent on sulfation pattern; changing the position of a single sulfate altered the binding orientation of the CS fragment. The GAG-binding region of CCL5 overlaps with the region required for CCL5 to bind its receptor CCR1, as CCR1 binding is mediated through the CCL5 residues Arg17 and Arg47. Thus, long heparin fragments that include the most common heparin structural motif of the repeating trisulfated disaccharide and fragments as short as a tetrasaccharide, if they are of an appropriate structure, have been shown to block CCL5 from interacting with CCR1 [17]. Recently the solution structure of a doubly sulfated *N*-terminal fragment of CCR5 bound to CCL5 was determined and the structure revealed the important CCL5 residues for binding are Ile15, Arg17, Leu19, His23, Lys45, Arg47, and Val49 [38]. Given the similarity in residues involved CCR5 binding and GAG binding, appropriate GAG structures should also block CCL5-CCR5 interactions. It is likely that CS fragments of the appropriate structure, may similarly block CCL5-CCR1 and CCL5-CCR5 interactions. Moreover, the data are consistent with the view that the way GAGs influence CCL5 function is very much dependent upon their sulfation pattern.

CCL5 displays a marked propensity to oligomerize into higher order complexes at high pH or high concentrations, or in the presence of immobilised GAG/heparin [12]. It has been proposed from the molecular modelling of a “typical” CCL chemokine dimer interacting with a chemokine receptor that only CCL chemokine monomers are able to bind their receptors [12]. If so, as CCL5 dimers bind GAGs with higher affinity and stability than the monomer, and as GAG binding facilitates oligomerization, it could be argued that GAGs inhibit CCL5 signalling not because their binding site overlaps with the receptor binding site but because they facilitate oligomerization. However, this does not fit with the heparin tetrasaccharide study, as the tetrasaccharide structures that best inhibited CCL5 receptor binding were also those tetrasaccharides that not only occupied the ^44^RKNR^47^ motif but also used Arg17 for their binding [17]. In vivo, given the probable concentrations of CCL5 at the endothelial surface during inflammation, monomer numbers are likely to be low because the dimerization constant of CCL5 is in the nanomolar range [37]. Clearly, the manner in which CCL5 binds its receptors and the effects of GAG binding on receptor binding and dimer/oligomer formation are still not completely understood.

**Figure 4:**
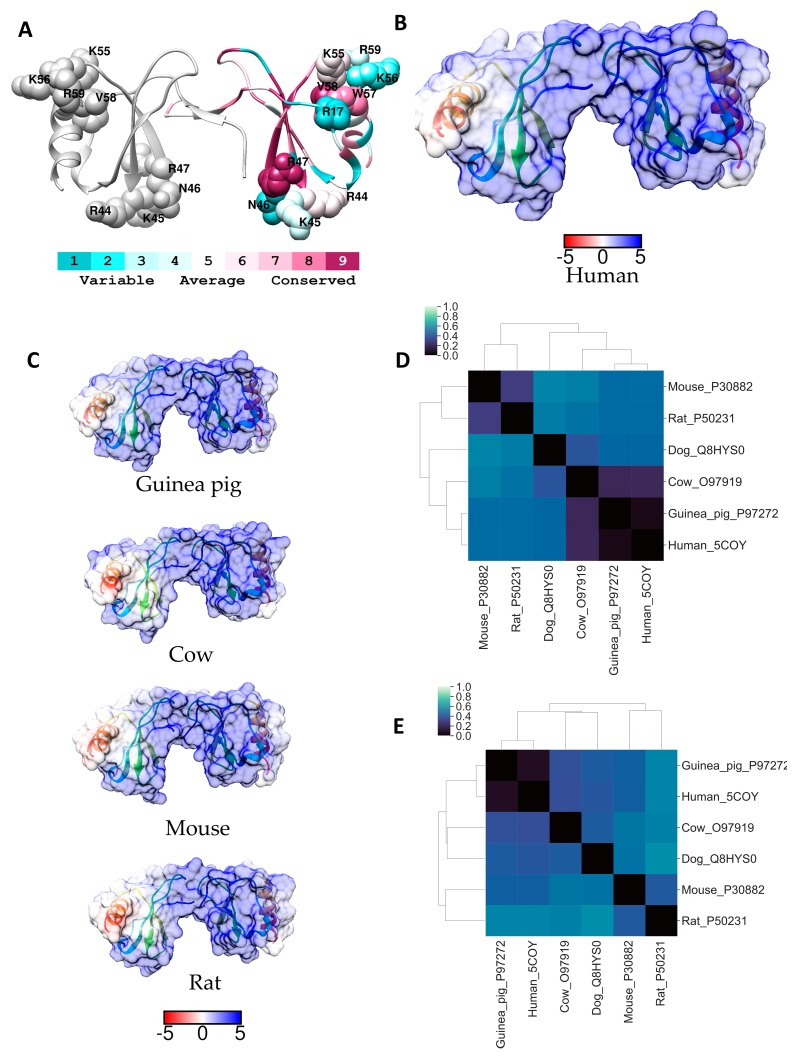
(**A**) Ribbon diagram of human CCL5 coloured according to Consurf conservation scores. GAG binding around the activate site have been labelled and depicted as spheres. Electrostatic surface potential (blue for positive and red for negative) shown for (**B**) human CCL5 and (**C**) for selected homologues of residues. Units for electrostatic potential are kT/e. Electrostatic difference (ESD) plot showing all-pairwise comparison of the electrostatic potentials for (**D**) CCL5 dimers (surfaces shown in (**B**) and (**C**)) and (**E**) monomeric CCL5.

A few different CCL5 oligomeric structures have been reported: a CCL5 tetramer derived from a hybrid method using NMR, SAXS, and molecular modelling (PDB code: 2L9H; pH 4.5); a double helical rod based CCL5(4–68) (PDB code: 5CMD, pH 7.5) and a CCL5(1-68) tetramer (PDB code: 2VXW; pH 5.5), and a double helical CCL5 20 mer and CCL5 hexamer (PDB code: 5DNF) with heparin octasaccharide at pH 7.5. The hybrid tetramer model suggested residues ^44^KKNR^47^ of the BBXB motif are essential for binding small GAG fragments with Lys55 and Lys56 contributing to a lesser extent [39]. However, the BBXB and the *N*-terminal GAG binding sites were largely buried in the higher order oligomeric structures and heparin octasaccharides were found to bind to the ^55^KKWVR^59^ motif [40]. This GAG binding motif had not been identified from analyses of CCL5 dimers with heparin at physiological pH, and it was suggested that the formation of CCL5 higher order oligomers was necessary to reveal this previously unknown GAG-binding site. Interestingly, a 2009 publication reported that the “50s” motif is required for immobilisation of CCL5 to endothelial cells and to tissue sections, and it is also required for macrophage recruitment to the peritoneal cavity in vivo [41]. Despite striking differences in the crystal structures of higher order CCL5 oligomers, and several theories on the mechanism of GAG/receptor interactions with CCL5, it is clear that ^44^RKNR^47^, Arg17, and ^55^KKWVR^59^ are surface exposed in the CCL5 dimer. We therefore performed Consurf (Figure 4A) and ESD analysis on the entire surface of the CCL5 dimer and the monomer (see Figure 4).

The hierarchical clustering phylogenetic analysis suggests that guinea pig CCL5 is the most similar to primate CCL5 (Figure 2). The heparin binding regions, BBXB motif ^44^RKNR^47^, plus Arg17, and the ^55^KKWVR^59^ motif are conserved in the human and guinea pig proteins (Figure S5). However, what is striking from the sequence alignment analysis is the lack of conservation of the ^44^RKNR^47^ motif in CCL5 from other species. The Lys45 in the human protein is not conserved; diverging sequences have an arginine at this position which is usually associated with increased affinity for GAG interactions. Whereas, Asn46 in the human CCL5 shows little conservation across homology models and is substituted for basic residues in the dog, cat and horse models. Similarly, Arg17 is not conserved being replaced by a leucine in the rat and mouse proteins, and a glycine and a histidine respectively in the dog and cat proteins. The other *N*-terminal residues that contribute to GAG binding Ile15 and Leu19 are conserved in guinea pig CCL5, and whilst Leu19 is conserved in all the sequences examined Ile15 is not, being replaced by a leucine in the rat, mouse and cat proteins. The residue that is required for receptor binding, Arg47, is completely conserved in all species examined. Curiously the motif ^55^KKWVR^59^ is more conserved than the “40s motif” in that residues 55–58 are completely conserved, but Arg59 is not conserved and is replaced by glutamine in the rat and mouse proteins. Although polar, under physiological conditions, glutamine has a neutral net charge which may reduce the ability of GAGs and GAG mimetics to bind rat or mouse CCL5 hexamers via this region. A comparison of the electrostatic surfaces revealed marked differences between CCL5 from different species (Figure 4B,C). The ESD data, presented in Figure 4D,E, very convincingly show that the surfaces of the human CCL5 dimer and monomer are most similar to that of the guinea pig CCL5 dimer and monomer. The rodent CCL5 surfaces are the least similar to the human; the mouse monomer appears more similar than the rat to the human protein, but when dimerised there is little difference between CCL5 from these two rodents. Whereas, for the cow protein the electrostatic surface of the dimer is more similar to that of the human dimer than is the case when the monomer surfaces are compared. Despite the complexities of CCL5/receptor/GAG biology from these analyses it is clear that the guinea pig is the most appropriate of the commonly used laboratory animals to use for testing GAG mimetics targeting CCL5.

### 2.4. Eotaxin-1 (CCL11)

A chemokine that shares two receptors with CCL5 is eotaxin-1. Eotaxin-1, as well as binding its receptor CCR3, can also bind CCR2 and CCR5 but at a much lower affinity, up to two orders of magnitude less than its affinity for CCR3 [42]. Thus, CCR3 is known as the predominant receptor of eotaxin-1. The solution structure of monomeric eotaxin-1 bound to the *N*-terminal fragment of CCR3 has revealed that the sulfotyrosine residues of this receptor fragment bind to a crevice on eotaxin-1 comprising the *N*-loop and β2-β3 regions [43]. Interestingly, loss of sulfation reduces receptor activity by approximately 400-fold in the presence of eotaxin-1, and binding of the CCR3 *N*-terminal peptide to eotaxin-1 is enhanced as sulfation increases from a single sulfate to two sulfates. The positively charged side chain groups of Arg16 and Lys47 in eotaxin-1 were found to specifically interact with the sulfated groups on Tyr216 and Tyr217 from CCR3. Interestingly, it has been shown that the *N*-terminal sulfated fragment of CCR3 and a similar *N*-terminal sulfated fragment of CCR5 bind respectively eotaxin-1 and CCL5 by equivalent residues, and this is despite differences in sulfation pattern, orientation, and conformation of the receptor fragments [43].

**Figure 5:**
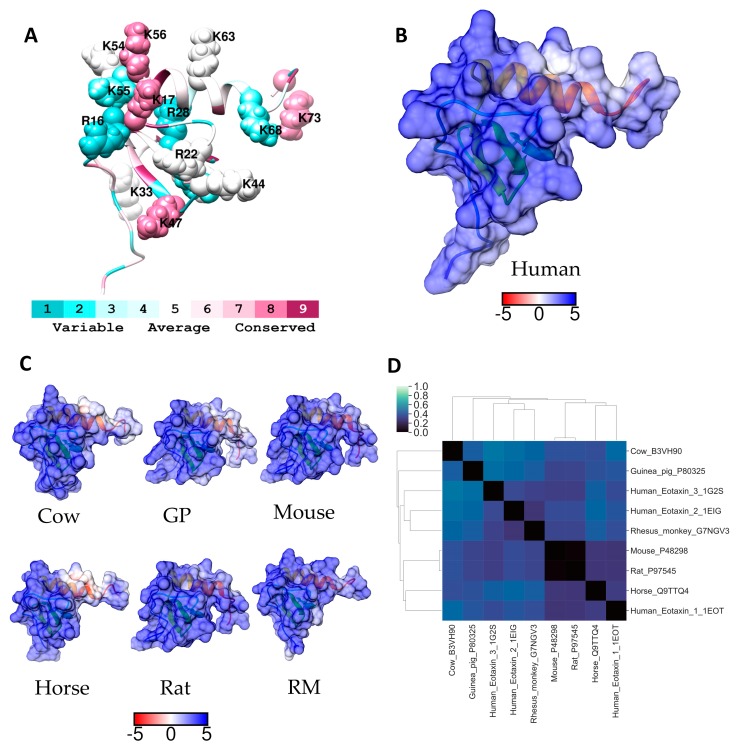
(**A**) Ribbon diagram of human eotaxin 1 coloured according to Consurf conservation scores. All basic residues have been labelled and depicted as spheres. Electrostatic surface potential (blue for positive and red for negative) shown for (**B**) human eotaxin 1 and (**C**) for selected homologues. Units for electrostatic potential are kT/e. (**D**) Electrostatic difference (ESD) plot showing all-pairwise comparison of the electrostatic potentials for surfaces shown in (**B**) and (**C**).

The eotaxin-1 sequence is the least conserved sequence among the proteins investigated. According to the clustering of the overall sequence similarity, primate eotaxin-1 is more closely related to the horse and cow proteins than to rodent eotaxin-1 (Figure 2). The closest homolog to the human sequence is the rhesus macaque (G7NGV3). While the rodent sequences (rat and mouse) share a high degree of similarity, overall sequence similarity to the human protein is low. While this is the case, there are several highly basic regions that are conserved in terms of their charge across all species, but the residues are not necessarily conserved (Figure S6). This is true for residues in the *N*-terminal loop; ^16^RK^17^ in the human and horse proteins, becomes ^16^KK^17^ in all other species examined. Arg22 in the human protein becomes, Leu22 in the rodent proteins, and Arg27 is replaced by a lysine in the rodent and guinea pig proteins. A striking motif in the primate, rodent and guinea pig proteins is the region ^54^KKK^57^ which is similar to the “50s motif” in CCL5. However, in the cow and horse this becomes respectively, ^54^QEK^57^ and ^54^KQK^57^. Another region could be considered as analogous to the “40s motif” described for CCL5. This is ^42^KTKLAK^47^ in human eotaxin-1. Of these residues Lys47 is completely conserved, Lys44 is replaced by an arginine in the mouse, and Lys42 is replaced by asparigine in the horse and glutamic acid in the guinea pig. The intervening non-basic residues at positions 45 and 46 are not conserved. A ribbon diagram showing the positions of the basic residues in the eotaxin-1 monomer is shown in Figure 5A. The corresponding electrostatic surface potential of the entire protein shown in Figure 5B indicates the highly basic nature of this protein.

Eotaxin-1 has variously been described as monomeric in solution, or existing in a monomer–dimer equilibrium under physiological conditions, but with monomer predominating at lower pH [44,45]. In contrast to many other chemokines, eotaxin-1 has been reported to retain its monomeric state when bound to heparin octasaccharides [44], and it exhibits an usually high preference to bind heparin over other types of GAGs, including HS [16]. More recently a tetrameric form was described and it was found both in the absence of GAG and bound to fondaparinux (Arixtra^TM^) [46]. However, to our knowledge, no confirmed crystal structure was deposited in the RCSB Protein Data Bank. Given the similarity of the basic regions in eotaxin-1 and CCL5 and the similarities in the way these chemokines bind their receptors we chose to concentrate on the eotaxin-1 monomer, accordingly the electrostatic surface potentials for the homologous proteins were calculated (see Figure 5C) and compared to that of the human protein. These data show that the *C*-terminal region of the helix is less basic in the guinea pig, cow, and horse eotaxin-1 compared to the human protein.

Since the GAG-eotaxin-1 complex is currently uncharacterized, ESD calculations were performed on the entire monomer. ESD calculations were also included for the other eotaxin family members, eotaxin-2 (CCL24) and eotaxin-3 (CCL26) from humans (Figure 5D). A comparison of the ESD calculations of these homologs suggests that rat and mouse eotaxin-1 are extremely similar and the surfaces of these proteins are the most similar to that of human eotaxin-1. These data are in contrast to the hierarchical clustering analysis based on percent identity (Figure 2). The ESD data suggest that rat or mouse models will be better than guinea pig, or rhesus macque models, for studying eotaxin-1. Whereas rhesus macque models appear better than rodent models for studying eotaxin-2. In some cases the surface potentials of eotaxin-2 and -3 were more similar to eotaxin-1 than available homology models, such as bovine eotaxin-1, but the surfaces of rodent and human eotaxin-1 were more closely related than that of the other human eotaxins. The sequences of the three eotaxins are aligned with the CCL5 sequence in Figure S7, and these data further emphasise the close relationship of the critical regions for receptor and GAG binding in these four related proteins.

### 2.5. IL-8 (CXCL8)

IL-8 belongs to the CXC chemokine family, having the CXC arrangement of cysteine residues that participate in two disulfide bonds that stabilize the tertiary fold of the three *β*-strands and single *α*-helical segment characteristic of this chemokine family. It exists in a dynamic equilibrium of monomers and dimers. IL-8 binds to two G-coupled-protein receptors, CXCR1 and CXCR2, to mediate neutrophil chemotaxis and activation. CXCR1 is specific for IL-8, whereas CXCR2 also binds other CXC chemokines: CXCL1, CXCL2, CXCL3, CXCL5, CXCL6, and CXCL7 [47]. IL-8 has a flexible *N*-terminal segment containing a conserved Glu-Leu-Arg (ELR) motif which interacts with the CXCR1 extracellular loop residues, whereas the IL-8 *N*-loop, encompassing residue Lys15, interacts with the *N*-terminal residues of CXCR1 [48,49]. IL-8 binds to CXCR2 in a similar manner, but IL-8 monomers bind CXCR1 with higher affinity than the dimer, whilst the difference between the affinities with which monomeric and dimeric IL-8 bind CXCR2 is much less [50]. The GAG binding site in IL-8 (*K*_D_ heparin = 0.4 – 2.6 μM) partially overlaps the “high-affinity” receptor binding domain [51]. NMR studies (^1^H–^15^N HSQC correlations) identified key CXCR1 binding regions in IL-8 as the *N*-loop motif: ^10^ITYSK^15^, Phe17, His18, Lys20, and Phe21, and adjacent β-strand residues Glu48 and Leu49 [52]. Residues Lys15, His18, Lys20, are common to the GAG binding region, which also includes Lys23 and helical residues Arg60, Lys64 and Arg68 [14,15]. Moreover, heparin bound IL-8 monomers or dimers are also impaired from binding their other receptor, CXCR2. Key IL-8 amino acids for CXCR2 binding are the *N*-loop motif ^10^ITYSK^15^ and His18 as well as β-strand residues Glu48 and Leu49. The overlap with the site required for CXCR1 binding and GAG binding is evident [25]. The data indicate that the IL-8 dimer binds GAGs with higher affinity than the monomer, which has led to the suggestion that dimers will exist primarily bound to GAGs, whilst monomers are either in the free form or bound to receptors. Thus, GAG interactions regulate IL-8 receptor binding and signalling and hence neutrophil migration [25].

A gene duplication event in the rodent lineage, caused the genes encoding IL-8 and its receptor CXCR1 to be deleted in the mouse [53]. Mouse keratinocyte-derived protein chemokine (KC), MIP-2, and LIX are the accepted mouse homologues, however, these chemokines were found to bind heparin differently, with KC associating and dissociating more rapidly from immobilized heparin than the other chemokines [54]. The differences in the binding kinetics were attributed to the low sequence homology (~66% identity) of KC and MIP-2. Given these factors, the use of murine models to assess the efficacy of GAG mimetics targeting IL-8 is not appropriate. To identify a species which could be used for preclinical assessments of IL-8 targeted GAG mimetics the sequence similarities of IL-8 from a range of mammals were examined.

**Figure 6:**
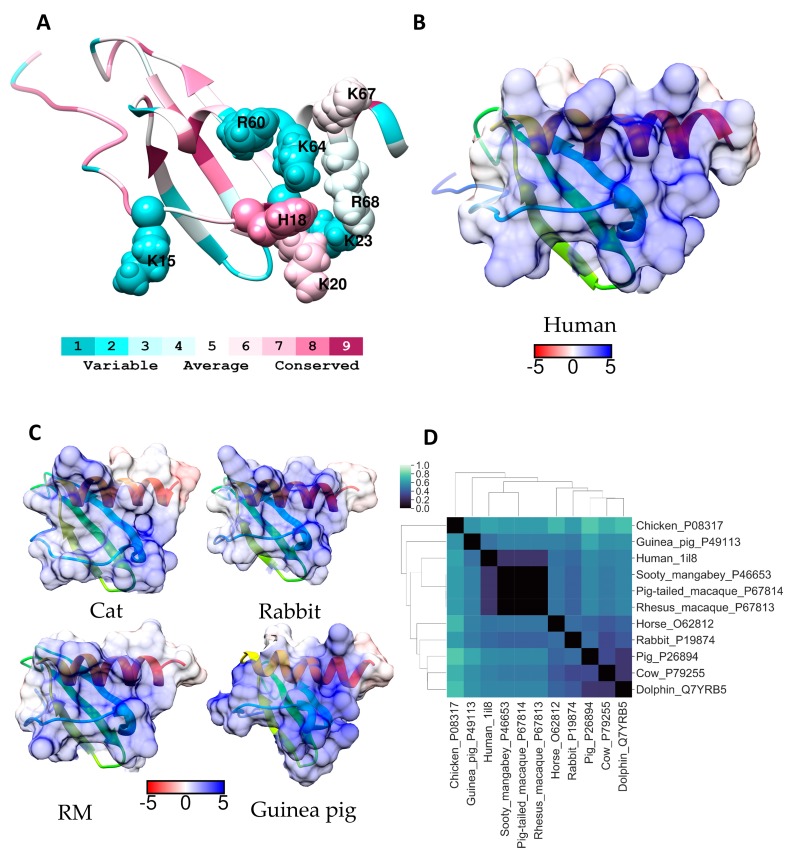
(**A**) Ribbon diagram of human IL-8 monomer coloured according to Consurf conservation scores. GAG binding around the activate site have been labelled and depicted as spheres. Electrostatic surface potential for the entire protein (blue for positive and red for negative) shown for (**B**) human IL-8 and (**C**) for selected homologues. Units for electrostatic potential are kT/e. (**D**) Electrostatic difference (ESD) plot showing all-pairwise comparison of the electrostatic potentials for surfaces shown in (**B**) and (**C**).

**Figure 7:**
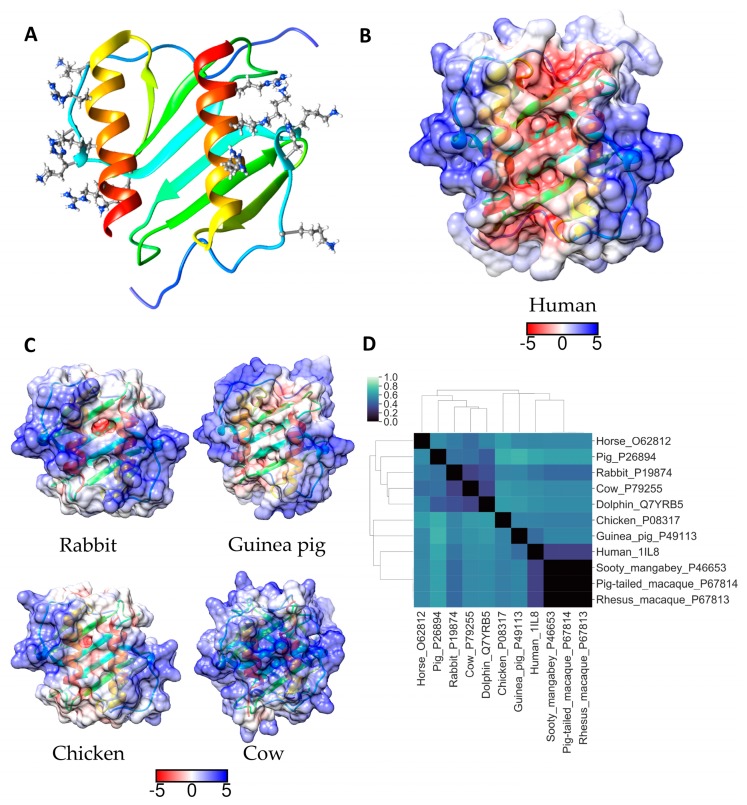
(**A**) Ribbon diagram of human IL-8 dimer coloured according to Consurf conservation scores. GAG binding around the activate site have been labelled and depicted as spheres. Electrostatic surface potential for the entire protein (blue for positive and red for negative) shown for (**B**) human IL-8 dimer and (**C**) for selected homologues. Units for electrostatic potential are kT/e. (**D**) Electrostatic difference (ESD) plot showing all-pairwise comparison of the electrostatic potentials for surfaces shown in (**B**) and (**C**).

The overall sequence similarity between IL-8 proteins is high between primate IL-8 and other small mammals such as rabbits and dogs. The basic residues that comprise the GAG binding site in IL-8 are on the opposite face to residues responsible for IL-8 oligomerization [54]. Consequently, the surface of the GAG binding site remains relatively unchanged between IL-8 monomers and dimers, and this is true for all the species examined (Figure S7). Lys20, Lys67, and Lys68 in the GAG binding site in human IL-8 are completely conserved in all of the mammals examined, and other GAG-binding site amino acids are highly conserved; Lys23 is conserved in all species with the exception of dolphin where it is replaced with an arginine, similarly His18 is conserved in all except horse and cat where it is replaced by an asparagine. In contrast, Arg60 is frequently replaced by a lysine, but in the guinea pig protein it is an aspartic acid and in the horse and chicken proteins an isoleucine and a leucine, respectively. Lys15 is present in primate IL-8 and the horse and chicken protein but in IL-8 from the other species it is a threonine, and Lys64 is highly variable being present only in primate IL-8 (Figure S7). In the ribbon diagram of monomeric and dimeric IL-8 it appears that Lys15 is on the fringe of the GAG binding site in human IL-8 (Figure 6A and Figure 7A) and may not be essential for GAG binding in some species. ESD calculations indicate the pattern of surface charge of human IL-8 is most like that of IL-8 from other primates and this is true for both monomers and dimers (Figure 6D and Figure 7D). The non-primate IL-8 homologues which are closest to human IL-8 in this parameter were from the rabbit and guinea pig. However, when the surface electrostatic potentials are visualised on these proteins, and on human IL-8, differences are apparent which are most clear in the dimer (Figure 6C and Figure 7B,C). Human dimeric IL-8 has a pronounced region of negative charge across the β-pleated sheets that separate the positively charged helical domains. In the rabbit, this is diminished and there is a weak positively charged region across the β-pleated sheets connecting the positively charged domains on the outer faces of the two helices (Figure 7B,C). Hence, it is probable that some GAG chains will bind to cross-link the two positively charged regions. Our preliminary molecular modelling supports this conclusion (data not shown). The electrostatic surface of bovine IL-8 dimers clearly suggests this alternative GAG binding mode will dominate for this IL-8 (Figure 7B,C) and it is therefore unlikely that GAG binding will regulate bovine IL-8 receptor interactions in the same manner as has been described for the human protein. In guinea pig and chicken IL-8 the positive charge is confined to the periphery of the dimer and GAG binding is likely to inhibit IL-8 engagement with its receptors as outlined for human IL-8. However, as the overall homology of the GAG-binding region in human and chicken IL-8 is less than for the guinea pig sequence, guinea pigs are proposed as the best laboratory animal for preclinical tests with GAG-mimetics targeting IL-8.

### 2.6. PF4 (CXCL4)

**Figure 8:**
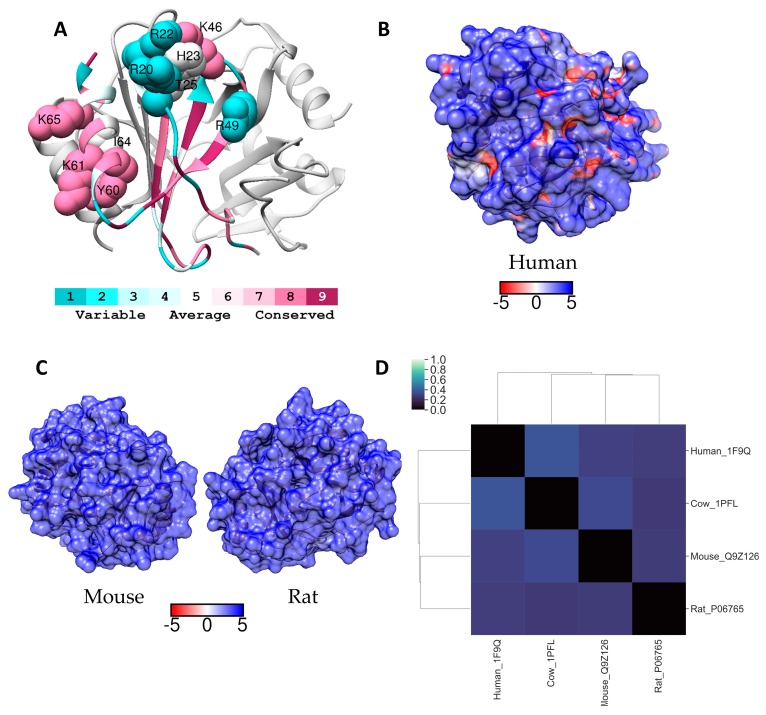
(**A**) Ribbon diagram of human PF4 coloured according to Consurf conservation scores. GAG binding around the activate site have been labelled and depicted as spheres. Electrostatic surface potential of the entire protein (blue for positive and red for negative) shown for (**B**) human PF4 and (**C**) for selected homologues. Units for electrostatic potential are kT/e. (**D**) Electrostatic difference (ESD) plot showing all-pairwise comparison of the electrostatic potentials for surfaces shown in (**B**) and (**C**).

Native human PF4 exists in a pH dependent equilibrium of monomers, dimers and tetramers with monomers being dominant at pH below 4 and as the pH increases tetramer concentrations increase at the expense of monomers. Tetramers form by the association of two dimers, but under physiological conditions tetramers predominate and there is very little dimer [55]. The tetramers are asymmetric in that the orientation of each of the monomers within the tetramer is slightly different giving a final structure where the basic residues cluster to form a positively charged ring around the PF4 tetramer (Figure 8A and [56]). Amino acids involved in heparin/GAG binding were found to be ^20^RPRH^23^ and of critical importance are Arg20 and Arg22 in the N-terminal helical loop region, the region comprising the motif ^61^KKIIKK^66^ in the C-terminal α-helix, and the region ^46^KNGR^49^ [57]. Originally, heparin or heparan sulfate were believed to wrap around these residues and a fragment of approximately 9 kDa was required for high affinity binding [58]. More recently, others have examined the binding of long and short heparin fragments. Short fragments do bind and the crystal structure of fondaparinux bound to PF4 has been published [26,27]. However, other data suggested that short fragments, < 8 saccharides, bind to one PF4 tetramer but long fragments bind to two or more PF4 tetramers and as the heparin cross-linked PF4 tetramers get close to each other hydrophobic interactions between the PF4 tetramers contribute to stabilising the formation of heparin-PF4 complexes. The conformation of PF4 bound to long heparin was found to differ from that of PF4 bound to short heparin fragments [59]. Heparin fragments ≥ 11 saccharides in length were shown to be necessary to produce the conformational change in PF4 structure that exposes the antigenic epitopes necessary to trigger heparin induced thrombocytopenia (HIT) [60]. Mouse models of HIT have been developed but, although antibodies to mouse PF4-heparin complexes form, thrombocytopenia and thrombosis do not occur as is the case in human HIT, probably because mice lack expression of the platelet receptor FcγRIIa which is needed for IgG dependent platelet activation [61]. Given these findings, why the predominately pentasaccharide heparin mimetic, PI-88, triggered HIT in clinical trials is unclear, unless the density of sulfation of this heparin mimetic was a contributing factor, as multiple binding events between the sulfated saccharides and PF4 are required for aggregate formation. Nevertheless, this puzzle prompted us to examine the GAG-binding sites on PF4 from non-human species to determine if preclinical models that better translate to the human situation are possible.

The overall sequence similarity between human PF4 and homologs is rather low when compared to the other GAG binding proteins examined in this manuscript, but this lack of conservation does not extend to the amino acids of the GAG-binding site. Krauel et al., examined heparin binding in PF4 from eight mammals, including most of the species shown in Figure S8, and they concluded that these residues are highly conserved [56]. Our analysis using Consurf conservation scores suggested that the *N*-terminal GAG-binding region was the least conserved (Figure S8) and this is particularly true for Arg20, which in sheep, rat and murine PF4 is transposed to a histidine, whereas it is a serine and asparagine respectively in porcine and bovine PF4 (Figure S8). In contrast, Arg22 retains its basic character but is either an arginine or a lysine and His23 becomes an arginine in rat PF4. The lysines that comprise the GAG-binding site are conserved with the exception of Lys66, which in sheep PF4 is an arginine. However, Arg49 in human PF4 is not conserved, becoming a histidine and a serine in porcine and rat PF4 respectively (Figure S8). Histidines can make important contributions to GAG binding and particularly so at slightly acid pH, as when the pH is 6.5 around half the histidines are protonated. Thus, we are in agreement with the earlier publication.

When comparing the surfaces of tetrameric PF4, differences in electrostatic potential of the human protein and that of the rat and mouse proteins are evident (Figure 8B,C). Whilst the rodent PF4 tetramers appear to be uniformly basic across their exposed surfaces, human PF4 retains small grooves of negative charge. The ESD calculations suggest that the rat homologue may be marginally more similar to human PF4 than the murine homolog (Figure 8D). The hierarchical clustering analysis similarly indicates that the rodent PF4 sequence is more similar to the human than PF4 from the other species we examined. Although our data suggests that the surface of human PF4 is less basic than the surfaces of the rodent homologs, these species should be acceptable for preclinical studies of GAG mimetics providing the possibility is taken into account that binding affinities for the rodent proteins may be slightly higher than seen with human PF4. The Krauel et al. study reports that rabbit and human PF4 have a total amino acid similarity of 81% and the basic residues involved in binding GAGs in these two proteins are totally conserved [56]. These data similarly suggest that the rabbit is likely to be an excellent model species for assessing the extent to which a GAG mimetic will trigger the production of anti-PF4-GAG mimetic antibodies.

In contrast to the other proteins examined in this study, PF4 binds a range of anionic species of different structures, for example the lipid A moiety on LPS, various nucleic acids including aptamers and polyphosphate chains, all of which have the potential to induce the exposure of neoepitopes in PF4 and trigger the production of anti-PF4-anionic species antibodies [56,62,63]. Thus, small differences in the amino acids in and around the GAG-binding site are unlikely to alter the potential for the anionic moiety to expose neoepitopes on PF4. Rather the critical feature seems to be PF4 aggregation and this is dependent upon the size of the anionic moiety and the density of its negative charges. This leads to the conclusion that a variety of laboratory animals (mice, rats, and rabbits) are likely to be suitable model species in which to assess GAG mimetic dependent anti-PF4 antibody production.

## 3. Methods

### 3.1. Sequence Selection

Crystal structures for the human GAG binding proteins analyzed in this study were retrieved from the Protein Data Bank [64]. The ConSeq [65] and ConSurf [66] servers were used to identify and align homologous sequences. Firstly, a BLAST search of the SwissProt database was performed, followed by a sequence alignment to the human protein using a MUSCLE alignment. A crystal structure of human AT (PDB code: 1azx) was selected as the template structure for sequence conservation analysis and homology modelling. Ape (Q5R5A3), cow (P413161), mouse (P32261), and sheep (P32262) sequences were retrieved and used to generate homology models and calculate ESD. The rabbit sequence of AT was predicted from cDNA as previously published [30]. Human heparanase (PDB code: 5E9C) was selected as the template structure for sequence conservation analysis and homology modelling. Chicken (Q90YK5), cow (Q9MYY0), mouse (Q6YGZ1), rat (Q71RP1) model sequences were retrieved and analyzed. Human CCL5 (PDB code: 5COY) was selected as the template structure for sequence conservation analysis and homology modelling. Cat (Q8SQ40), cotton rat (Q91ZL1), cow (O97919), dog (Q8HYS0), guinea pig (P97272), horse (Q8MKD0), mouse (P30882), rat (P50231), and rhesus macaque (Q8HYQ1) model sequences were retrieved and used to generate figures and calculate ESD. Human eotaxin-1 (PDB code: 1EOT) was selected as the template structure for sequence conservation analysis and homology modelling. Cow (B3VH90), guinea pig (P80325), horse (Q9TTQ4), mouse (P48298), rat (P97545), and rhesus macaque (G7NGV3) sequences were retrieved, as well as solution structures of eotaxin-2 (PDB code: 1EIG) and eotaxin-3 (PDB code: 1G2S). Although IL-8 (CXCL8) exists in two isoforms, IL-8_72_ and IL-8_77_, both are known to induce equipotent inflammatory responses in vivo [67]. The sequence and structural analysis for IL-8 were based on the 72 amino acid isoform (PDB code: 1IL8) due to the availability of crystal and NMR structures and experimental data on GAG-IL-8_72_ interactions. Rabbit (P19874), chicken (P08317), cat (Q9XSX5), dolphin (Q7YRB5), rhesus macaque (P67813), sooty mangaby (P46653), pig-tailed macaque (P67814), sheep (P36925), dog (P41324), cow (P79255), sheep (P26894), horse (O62812), guinea pig (P49113), and armadillo (Q102R3) were retrieved and used to generate figures and calculate ESD. Human PF4 (PDB code: 1F9Q) was selected as the template structure for sequence conservation analysis and homology modelling. Mouse (Q9Z126), pig (P30034), and rat (P06765) model sequences were retrieved. For bovine PF4, a crystal structure was available (PDB code: 1PFL) and was used in further analyses.

### 3.2. Phylogenetic Analysis and Clustering

Multiple sequence alignment of all proteins selected in the previous step was achieved using the Clustal Omega server, following the standard protocol [68]. The percent identity matrix obtained from this method was used in clustering analyses. Hierarchical clustering, using the nearest point algorithm in the Scipy library in the Python programming language was used to create the dendrogram in Figure 2.

### 3.3. Homology Modelling

Homology models in different species were retrieved from SwissModel through the Protein Model Portal (PMP) [69]. Only homology models made using the selected human crystal structures as a template, and with a model reliability rating of “A” on the PMP, were selected. For annotated sequences which were not available in the PMP, the modelling using the SwissModel server was performed, using the human 3D structure as a template for modelling. Structure alignment was achieved using the “MatchMaker” tool in UCSF Chimera [70]. For each protein, all structures and homology models were truncated to the same size of the shortest sequence to ensure a consistent comparison between species in the following electrostatic difference calculations.

### 3.4. Electrostatic Potential Surface Difference Calculations of GAG Binding Sites

Electrostatic difference calculations were performed using the AESOP Python framework [71], based on previously published methods [22] at 298.15 K. In this step, a solvent dielectric constant of 78.0 was chosen. The protein dielectric was set to 2.0. The dielectric boundary of the protein was defined by the molecular surface of a probe of 1.4 Å radius. A grid spacing of 1.0 Å was used. Before this step, the protonation states of structures were set using the PROPKA method in PDB2PQR [72]. Structures were then minimized and refined using Modeler [73]. Electrostatic surfaces were generated using PDB2PQR and APBS, using the Amber forcefield, at 298 K in water at pH 7.4, aside from heparanase which was modelled at pH 5.5.

To get a clearer indication of the conservation of GAG binding sites, ESD calculations of large proteins (AT and heparanase) were restricted to the surface around residues within 5 Å of known GAG binding residues (reported in Table 1, Figure 1, and Figure 3). The residues that constitute the binding sites for AT and heparanase were defined based on interactions reported in the literature and PDBsum [74]. For the smaller proteins (CCL5, IL-8, eotaxins, and PF4), receptor and GAG binding sites take up a significant proportion of the structure. Based on this, ESD calculations were performed on the entire structures. 

## 4. Conclusions

Increasingly, it is being understood that the choice of animal species to use for preclinical testing of drug candidates is of critical importance, as an inappropriate choice can have a major impact on the relevance of the data to the clinical situation. This is equally true for the development of GAG mimetics as drugs as it is for the development of small molecules as drugs. In both cases a lack of conservation of the drug binding site on the target protein in the species selected could to lead to inappropriate efficacy data. A further concern for GAG mimetics delivered systemically is the likelihood that the mimetic will trigger drug induced thrombocytopenia or anticoagulation. The molecular basis for both has been extensively researched and the proteins bound by GAGs/heparin in these pathways are known. This can place the GAG mimetic at an advantage over a small molecule competitor in that likely toxicity pathways are known for the mimetic, which is frequently not the case for a small molecule drug under development. However, like the binding sites targeted by some small molecules, not all GAG binding sites are conserved across the different mammalian species that are commonly used in drug discovery programs.

The technological advancements of recent years and the associated greater understanding of the nature of GAG binding sites has made it possible to assess protein targets of GAG mimetics from different species for the similarity of their GAG binding sites. The combinatorial approach described here of sequence alignment, molecular modelling and an assessment of surface potential differences around identified GAG binding sites in the human proteins will allow informed choices to be made as to the most appropriate species to be used in preclinical testing for the GAG mimetic under study. Importantly, the described approach is effective when knowledge of target protein receptor interactions, target protein GAG binding and target protein oligomerization status has directed the modelling and the area assessed for surface potential difference calculations. It is the combination that produces functionally relevant information. In some cases, the conclusion may be that a variety of animal models are equally relevant, or, in contrast, a careful choice is essential. We found targeting PF4 is an example of the former, whereas CCL5 as the target, is an example of the latter. Our approach seems to be equally relevant when the GAG binding site is a cleft and when it is an extended surface, for example consider the protein targets, heparanase and IL-8 respectively. Incorporation of this combinatorial approach into the decision making which leads to the choice of preclinical models should lead to data that is more readily translated into the clinical setting and as a consequence reduce the number of animal studies required.

## Figures and Tables

**Table 1:** Summary of the proteins and respective GAG binding sites investigated in this study.

Protein	GAG Binding Residues	Other Interactions	Ref.
Antithrombin (AT)	^46^RR^47^ K^136 235^RK^236^K^275 121^FF^122^ K^125^R^l29 132^RK^133 228^K	Thrombin	[6]
Heparanase	^389^G ^64^N ^391^Y ^97^T ^62^N ^224^N E^225^ E^343^ Q^270^ R^272 349^GG^350^	PI-88 and PG545 (GAG mimetics)	[24]
RANTES (CCL5)	^17^R ^44^RKNR^47 55^KKWVR^59^	CCR1, CCR3, CCR5, oligomerization	[17]
Eotaxin-1 (CCL11)	^44^KLAK^47 54^KKK^56^	CCR3	[^a^]
IL-8 (CXCL8)	^15^K^18^H^20^K^23^K^60^R^64^K^68^R	CXCR1, oligomerization	[25]
PF4 (CXCL4)	^20^R^22^PR^23 25^T ^46^K^49^R ^60^YK^61 64^IK^65^	CXCR3B, oligomerization	[26,27]

^a^ Putative GAG binding residues based on homology with RANTES (CCL5) presented in Figure S6.

## Supplementary Materials

The following are available online, Figure S1: The structure of human AT with fondaparinux, including hydrophobic surface representation and detailed interactions as obtained from PDBsum, Figure S2: Sequence alignment of human AT (PDB code: 1AZX) and homologs, coloured by Consurf conservation, Figure S3: The structure of human heparanase with a heparin tetrasaccharide, including hydrophobic surface representation and detailed interactions as obtained from PDBsum, Figure S4: Sequence alignment of human heparanase (PDB code: 5e9c) and homologs coloured by Consurf conservation, Figure S5: Sequence alignment of human CCL5 (PDB code: 5COY) and homologs coloured by Consurf conservation, Figure S6. Sequence alignment of human eotaxin 1 (PDB code: 1EOT) and homologs coloured by Consurf conservation, Figure S7: Sequence alignments of eotaxin 1, 2 and 3 with CCL5. Homologous residues between the CCR3 and GAG binding regions have been highlighted, Figure S8: Sequence alignment of IL-8 (PDB code: 1IL8) and homologs coloured by Consurf conservation, Figure S9: Sequence alignment of human PF4 (PDB code: 1F9Q) and homologs coloured by Consurf conservation.
