# Supplementary material for: Cross-Species Analysis of Glycosaminoglycan Binding Proteins Reveals Some Animal Models Are “More Equal” than Others
Source: Molecules. 2019 Mar 6;24(5):924. doi: 10.3390/molecules24050924 (PMC6429508; doi:10.3390/molecules24050924)
Supplement: Supplementary file 1 [file molecules-24-00924-s001.pdf]

Cross-Species Analysis of Glycosaminoglycan Binding Proteins Reveals Some Animal Models are "More Equal" than Others

Supporting Information

Eric D. Boittier [1], Neha S. Gandhi [2,*], Vito Ferro [1] and Deirdre R. Coombe [3,*]

[1] School of Chemistry and Molecular Biosciences, the University of Queensland, Brisbane, QLD 4072, Australia; eric.boittier@uq.net.au (E.D.B.); v.ferro@uq.edu.au (V.F.)
[2] School of Mathematical Sciences and Institute for Health and Biomedical Innovation, Faculty of Science and Engineering, Queensland University of Technology, Brisbane, QLD 4000-, Australia
[3] School of Pharmacy and Biomedical Sciences, Curtin Health Innovation Research Institute, Faculty of Health Sciences, Curtin University, Perth, WA 6102, Australia
* Correspondence: neha.gandhi@qut.edu.au (N.S.G.); d.coombe@curtin.edu.au; Tel.: +61-8-9266-9708 (D.R.C.)

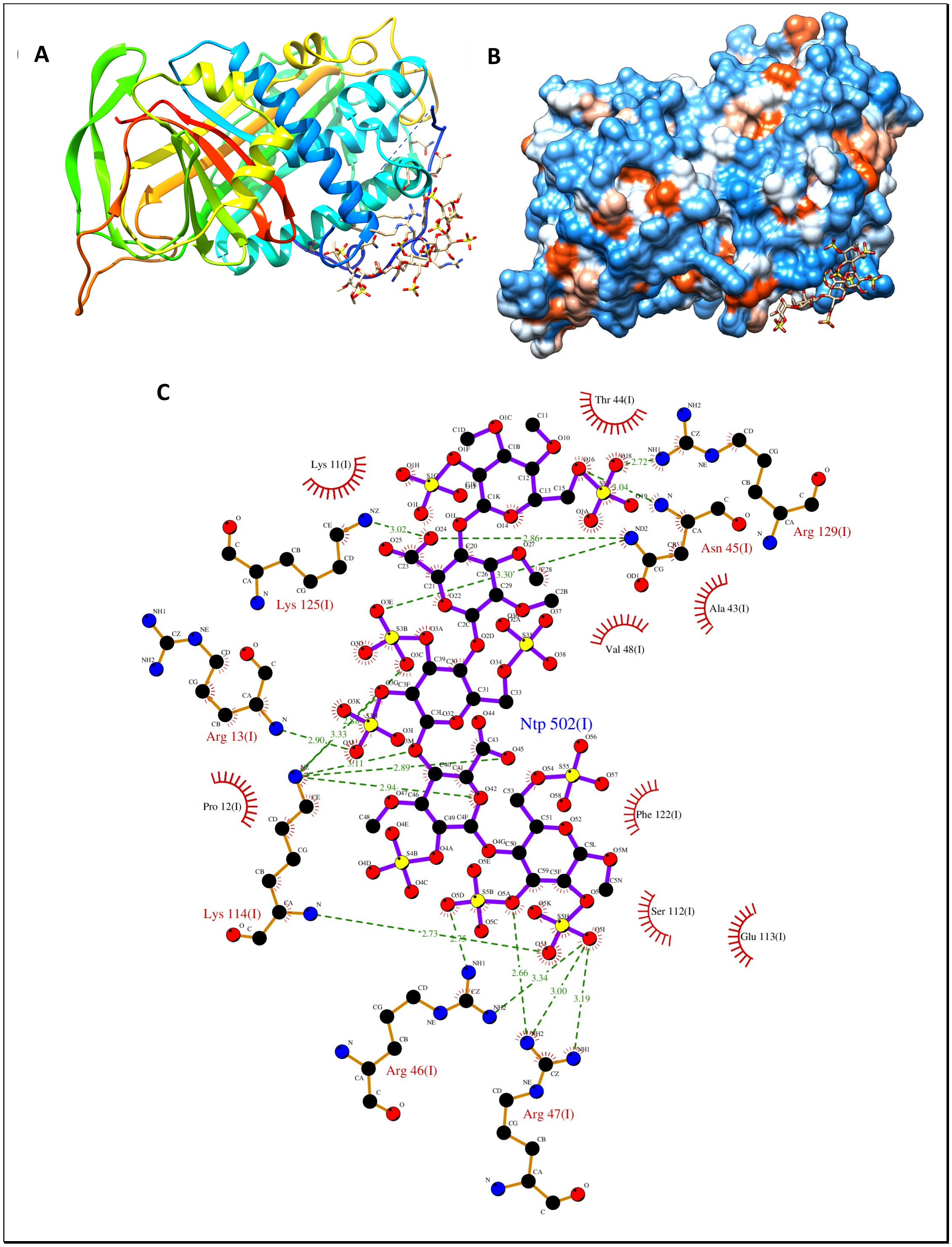

Figure S1. (**A**) Crystal structure of AT with fondaparinux. (**B**) The hydrophobic surface representation suggests that heparin pentasaccharide/fondaparinux binds on the surface of AT. The surface is presented in the same orientation as the ribbon diagram. (**C**) Details of interactions of fondaparinux with AT as obtained from PDBsum.

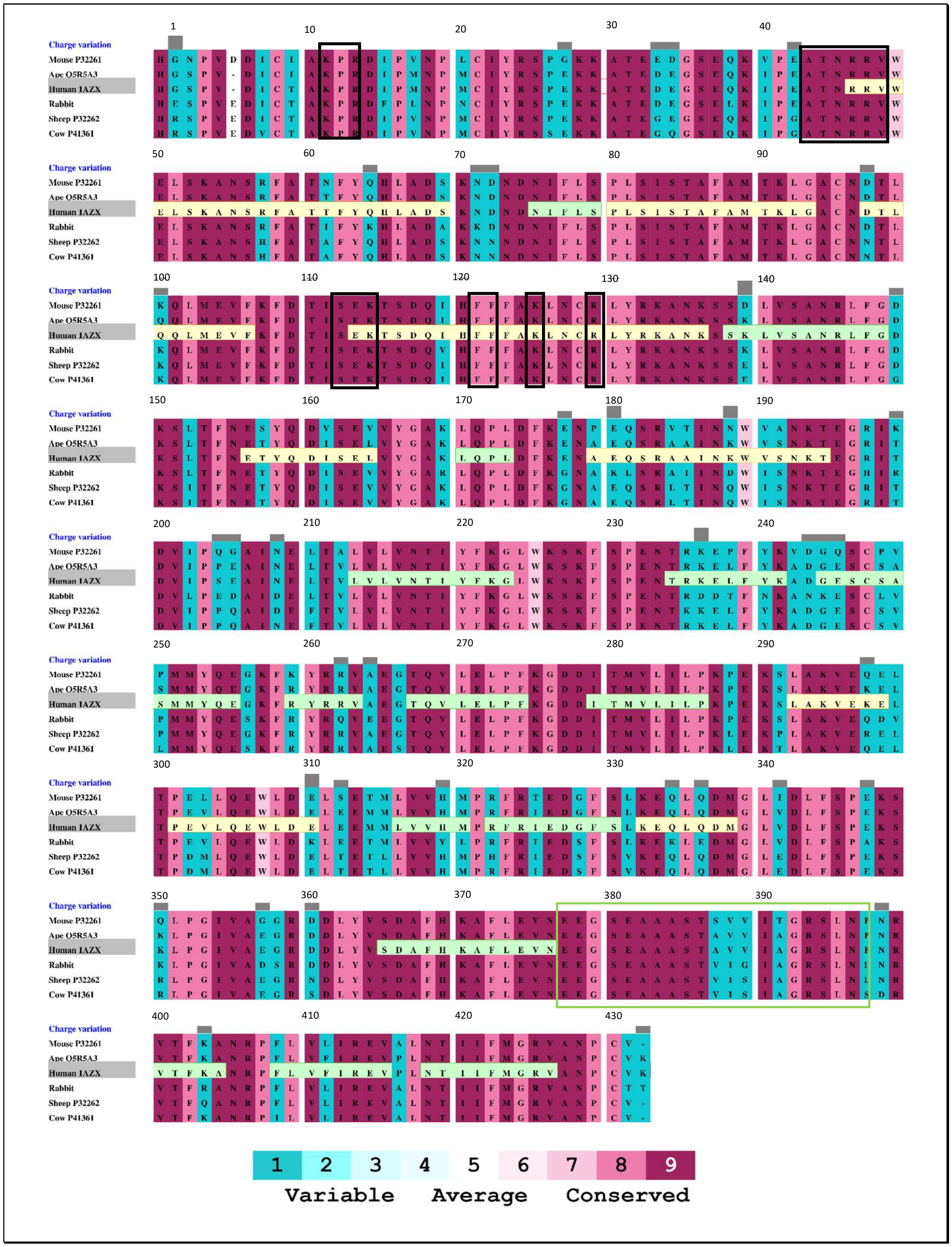

Figure S2. Sequence alignment of human AT (taken from pdb 1AZX) and homologs coloured by Consurf conservation. The secondary structure helix and beta is represented by yellow and green, respectively. The green box shows RCL binding site whereas the black box indicate residues in fondaparinux binding site.

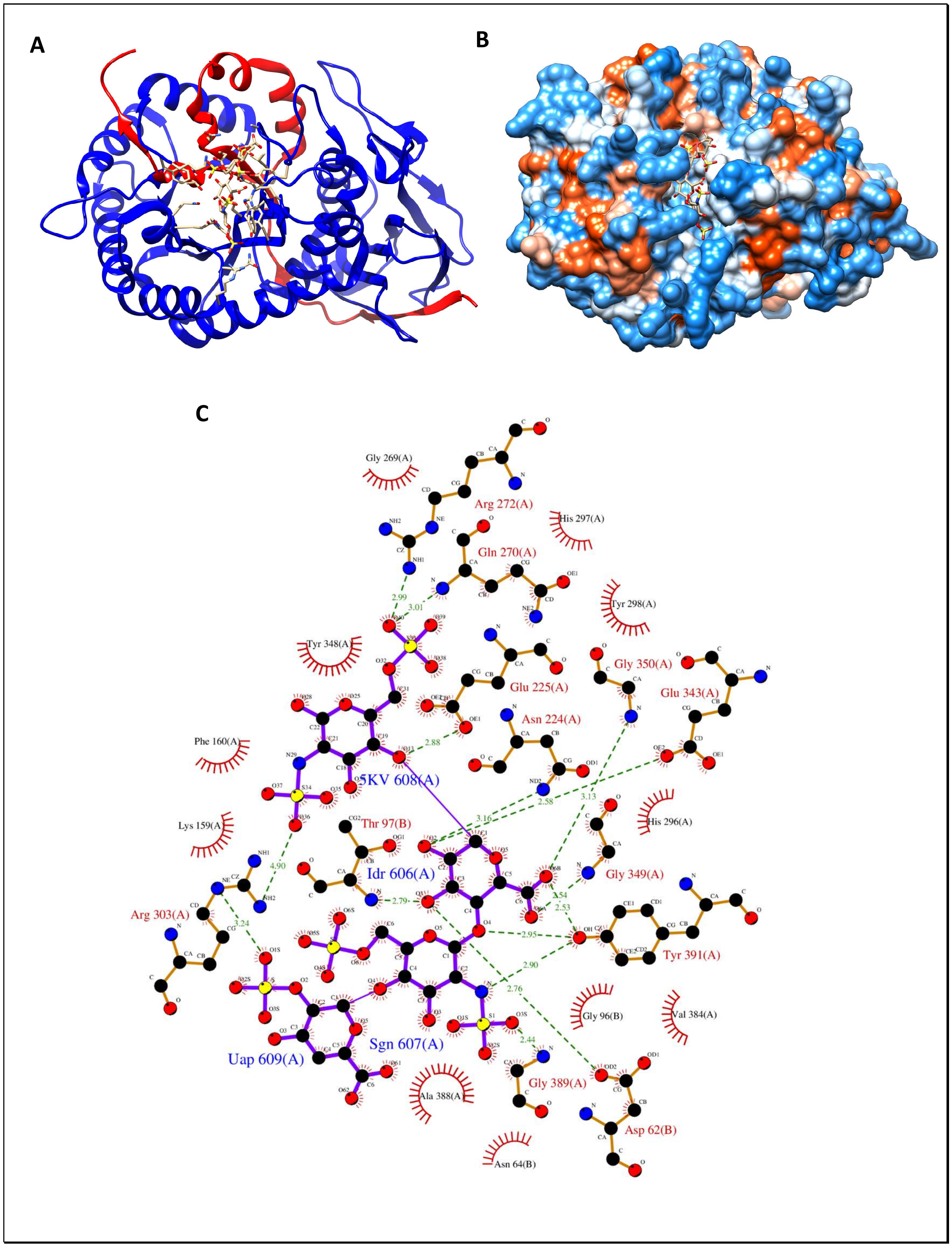

Figure S3. (**A**) Crystal structure of heparanase (two chains shown in red and blue) with heparin tetrasaccharide. (**B**) The hydrophobic surface representation suggest that heparin tetrasaccharide binds in a cavity of heparanase. The surface is presented in the same orientation as the ribbon diagram. (**C**) Details of interactions of heparin with heparanase as obtained from PDBsum for 5e9c structure.

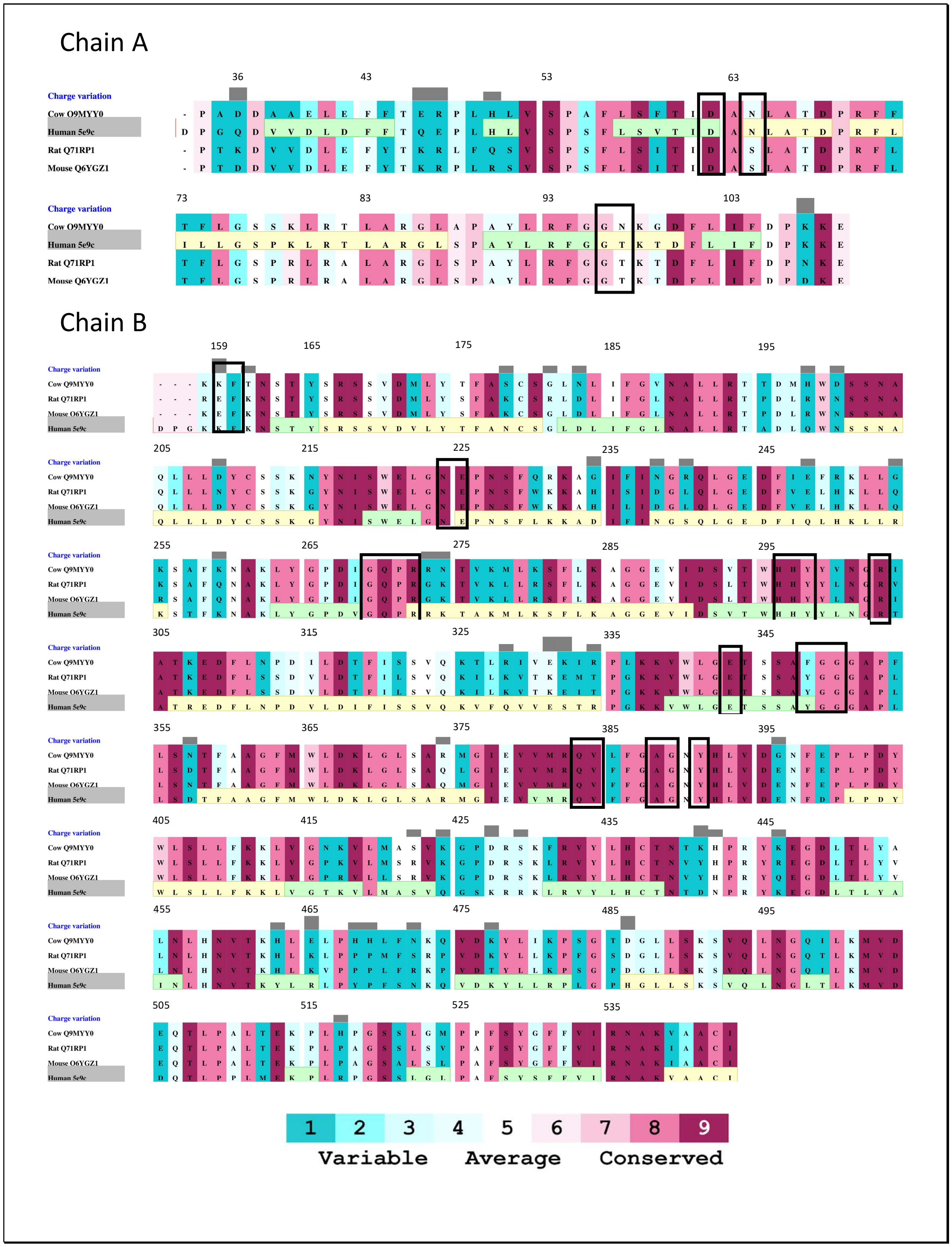

Figure S4. Sequence alignment of human heparanase (taken from pdb 5e9c) and homologues coloured by Consurf conservation. The secondary structure helix and beta is represented by yellow and green, respectively. The black box indicate residues in heparin binding site.

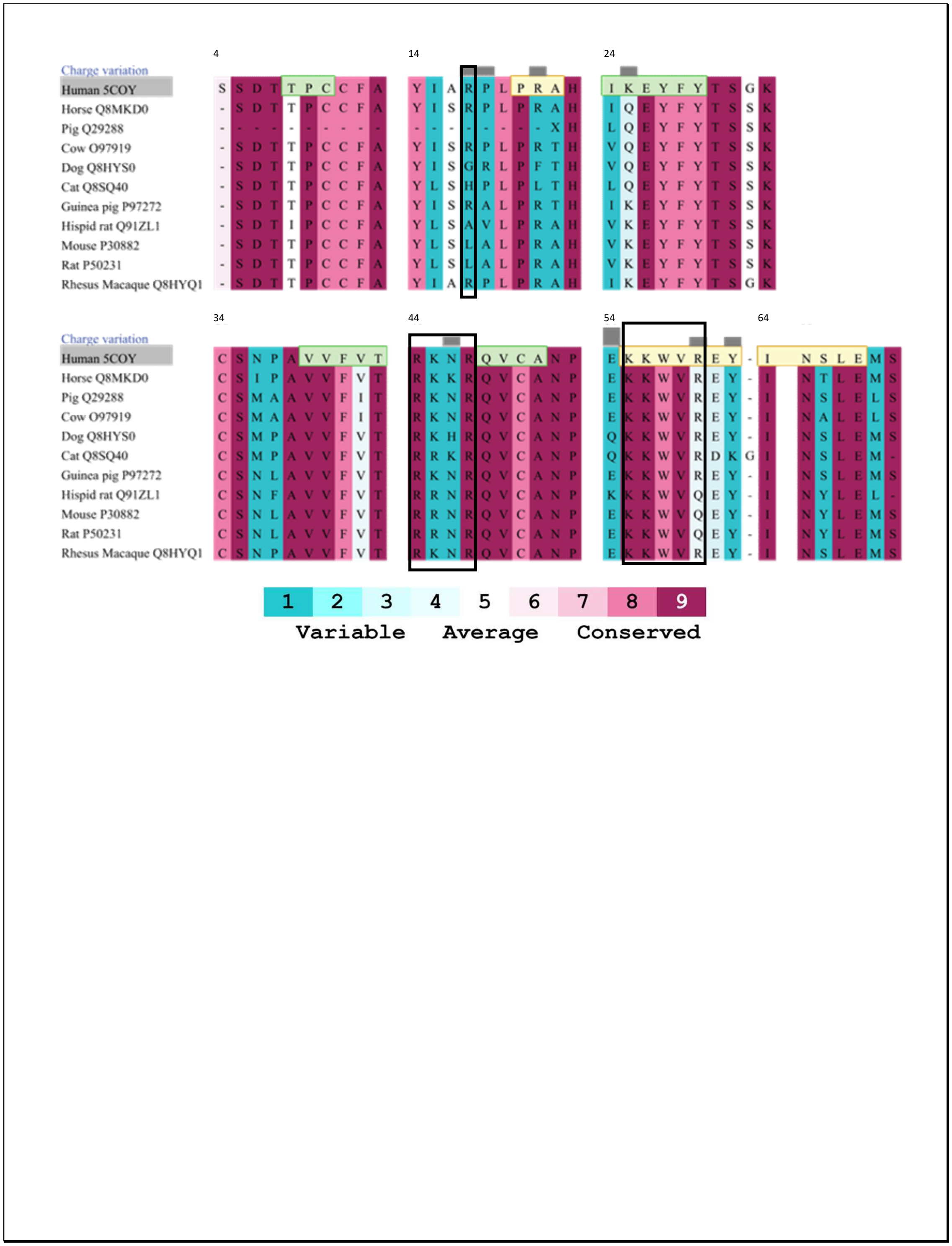

Figure S5. Sequence alignment of CCL5 (taken from pdb 5COY) and homologs coloured by Consurf conservation. The secondary structure helix and beta is represented by yellow and green, respectively. The black box indicate residues heparin binding site.

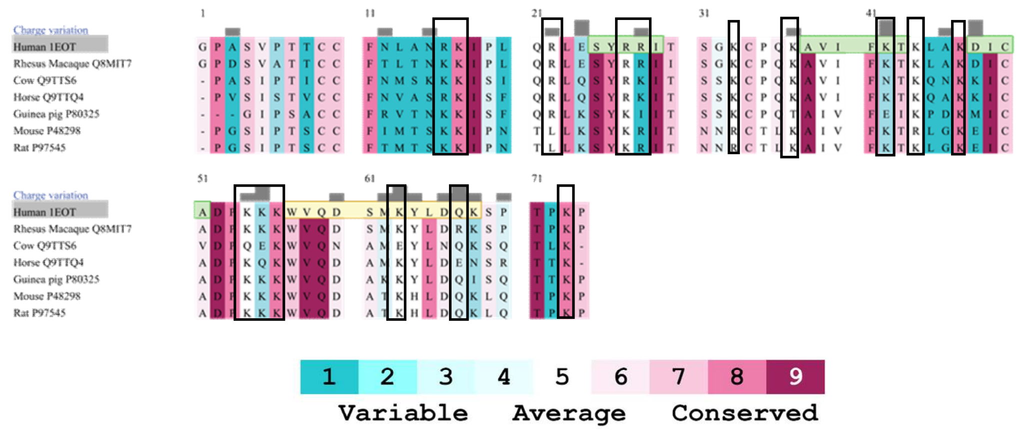

Figure S6. Sequence alignment of Eotaxin 1 (taken from pdb 1EOT) and homologs coloured by Consurf conservation. The secondary structure helix and beta is represented by yellow and green, respectively. The black box indicate residues heparin binding site.

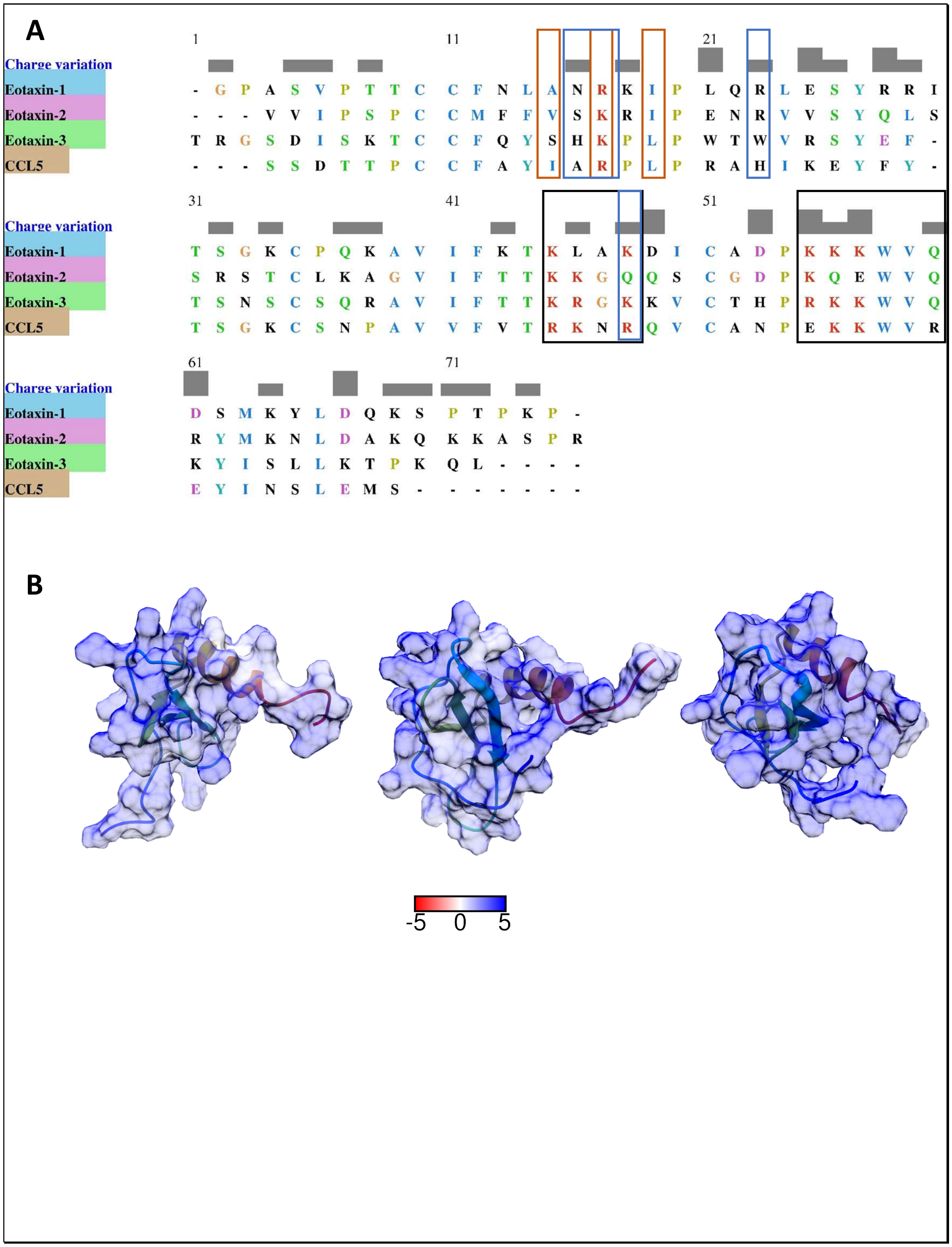

Figure S7. (A) Sequence alignments of eotaxin 1, 2 and 3 with CCL5. Eotaxin receptor, CCL5 receptor and CCL5 GAG binding residues are shown in blue, brown and black boxes, respectively. (B) Electrostatic surface potential surface representation for all three eotaxins.

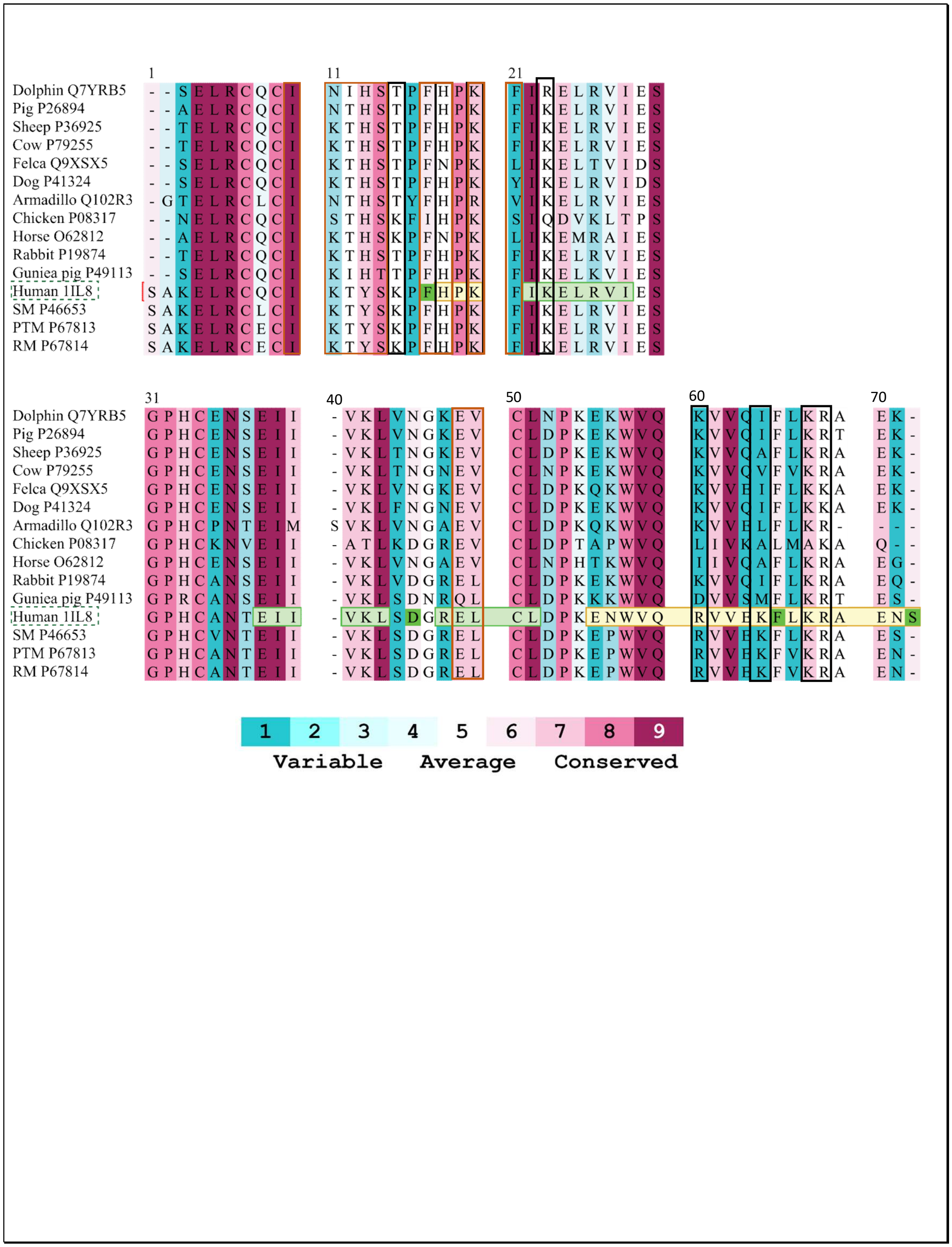

Figure S8. Sequence alignment of IL-8 (taken from pdb 1IL8) and homologs coloured by Consurf conservation. The secondary structure helix and beta is represented by yellow and green, respectively. The black and orange box indicate residues heparin binding site.

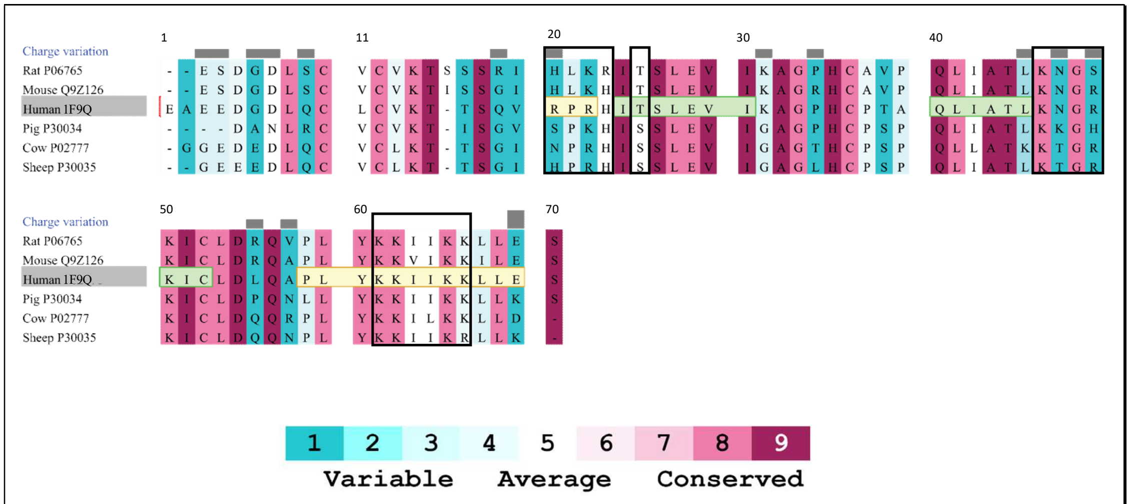

Figure S9. Sequence alignment of PF4 (taken from pdb 1IL8) and homologs coloured by Consurf conservation. The secondary structure helix and beta is represented by yellow and green, respectively. The black and orange box indicate residues heparin binding site.
